# Tuberous Breast, Deformities, and Asymmetries: A Retrospective Analysis Comparing Fat Grafting Versus Mastopexy and Breast Implants

**DOI:** 10.1007/s00266-022-03089-x

**Published:** 2022-09-26

**Authors:** Pietro Gentile

**Affiliations:** 1 Associate Professor of Plastic and Reconstructive Surgery, Department of Surgical Science, Tor Vergata” University, 00133 Rome, Italy; 2 Scientific Director of Academy of International Regenerative Medicine & Surgery Societies (AIRMESS), 1201 Geneva, Switzerland

**Keywords:** Tuberous breast, Breast asymmetry, Breast hypoplasia, Fat grafting, Breast implants, Tuberous breast correction, Plastic surgery

## Abstract

**Background:**

The tuberous breast is considered a breast deformity characterized by varying degrees of herniation of the parenchyma, widened nipple-areolar complex (NAC), absence of the lower quadrants, and may involve several degrees of hypoplasia and asymmetry causing significant psychosocial distress.

**Objectives:**

The paper aimed to compare the results obtained in patients suffering tuberous breast treated with fat grafting (FG), with those of patients treated with a mastopexy and silicone implants (M-SI) also analyzing the influence of breast and chest deformities (degrees of hypoplasia and tuberous breast, volume and NAC asymmetry, pectus excavatum, and carinatum) in the reconstructive outcomes.

**Methods:**

A retrospective, case-control study was conducted. Thirty-five patients affected by tuberous breast with several degrees of hypoplasia and asymmetry were treated with FG, comparing results with those of 30 patients treated with M-SI. Postoperative follow-up took place at 1, 3, 7, 12, 24, 48, weeks, and then annually for 2 years.

**Results:**

77% (*n* = 27) of patients treated with two FG procedures showed excellent results after 1 year compared with the patients treated with only one M-SI procedure, who showed the same results in 73% (*n *= 22) of cases, but the naturalness and the satisfaction degree in the FG group were higher than that in the M-SI group (*p *< .0001 *vs*. M-SI group).

**Conclusions:**

Patients treated with FG showed natural breasts without scars and excellent cosmetic results after two procedures. Patients treated with M-SI showed more evident and lasting results after only one procedure, presenting though scars and less natural results.

**Level of Evidence IV:**

This journal requires that authors assign a level of evidence to each article. For a full description of these evidence-based Medicine ratings, please refer to the Table of Contents or the online Instructions to Authors www.springer.com/00266

## Introduction

The tuberous breast deformity is considered a congenital asymmetry in which an alteration of the superficialis fascia, limits both the physiological expansion of the breast mound, producing hypoplasia, and thus its shape during growth outcoming in tuberosity [1–3]. According to Grolleau et al. [1], type I is a hypoplasia of the medial lower quadrant (LQ), type II is hypoplasia of both LQs, and type III is a hypoplasia of the four quadrants with severe breast constriction [1, 4]. As noted by Delay et al. [5], types II and III are the ones frequently treated as these present the more obvious and significant deformity. The exact tuberous breast’ incidence is unknown [6].

Several approaches have been described to correct the tuberous breast deformity starting with Rees and Aston [7] in 1976, with a gradual shifting, in the last years, from the mastopexy with silicone implants (M-SI) to less invasive strategies based on fat grafting (FG) [8]. FG has been also used for esthetical purposes with excellent outcomes [9]. A recent study compared the results obtained in breast hypoplasia treated with silicone implants (SI) with those obtained using FG enriched with Adipose-derived Mesenchymal Stem Cells (ASCs), confirming the safety and effectiveness of both procedures, showing however that FG allows decreased scar burden with natural esthetic results [10].

The significant side effects of SI use, like displacement, deformities, rejection, wrinkling, rippling, and the recent association with anaplastic lymphoma led to the development of noninvasive procedures [11]. Both the right degree’ analysis of tuberous breast and asymmetries, the evaluation of breast soft tissue, prevalently represented by muscle, gland, and fat that patient ‘expectations, plays a pivot role in the remodeling choice (SI, M-SI, or FG) [9, 11–13].

The present work aims to compare, the results obtained in tuberous breast and asymmetry correction using FG, with those obtained using M-SI, analyzing the influence of breast and chest deformities (asymmetry, hypoplasia, volume differences, and NAC asymmetry, pectus carinatum and excavatum) and the low body mass index (BMI).

## Methods

### Study Overview

A retrospective case-control study, classified as evidence-based medicine (EBM) level 3, was performed fully respecting the Declaration of Helsinki and internationally consented ethics in clinical research [14]. The Strengthening the Reporting of Observational Studies in Epidemiology (STROBE) checklist [15] was used to make a quality assessment.

### Patients

Over 15 years (2007–2022), 65 patients affected by breast deformity, breast hypoplasia, breast asymmetry, and several types of tuberous breast classified according to Grolleau et al. [1] were treated and divided into two groups.

**Fig. 1:**
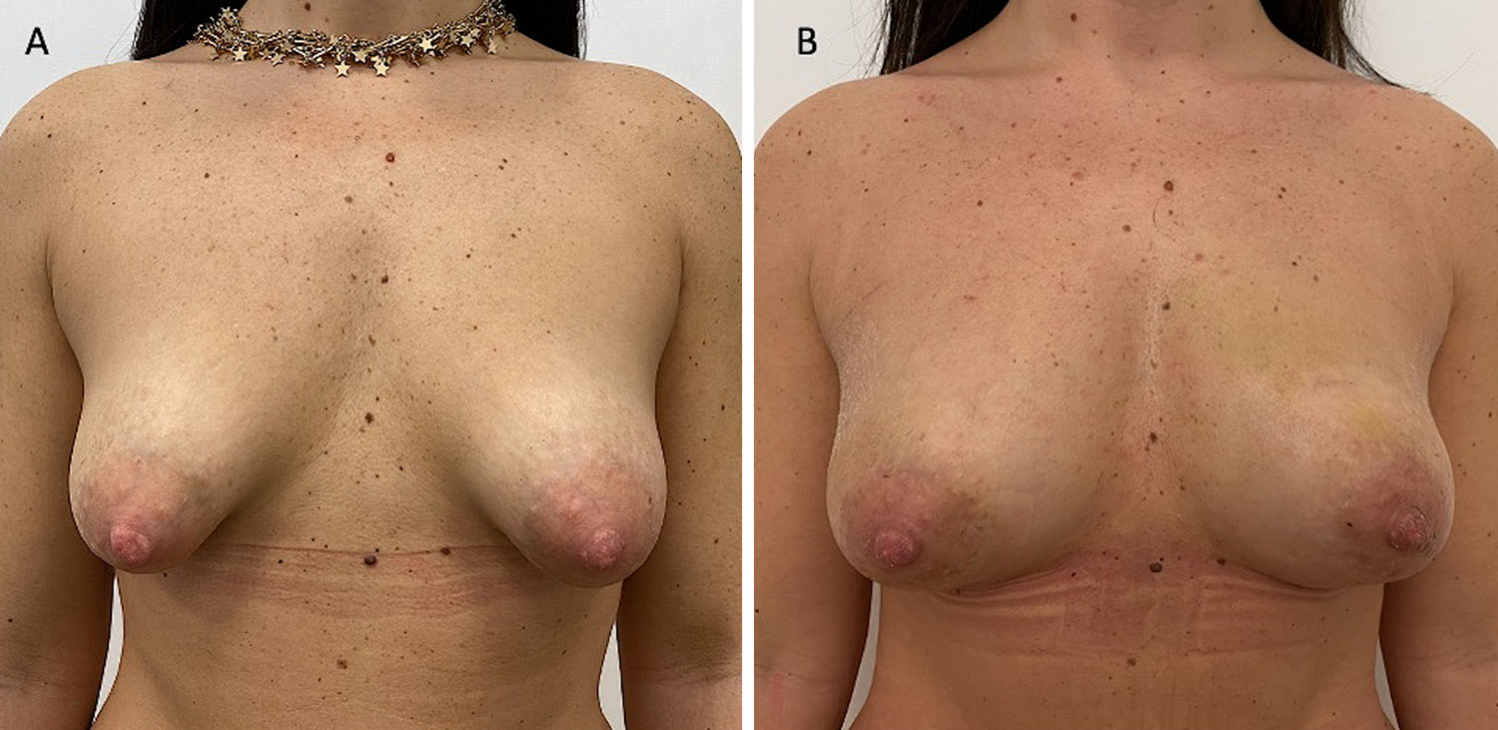
Analysis of the study group’s patient, a 33-year-old female patient affected by a bilateral tuberous breast of type II and bilateral hypoplasia. **A** Frontal preoperative view showing hypoplasia of the four quadrants with severe breast constriction; **B** Postoperative in frontal view 12 months later only one fat grafting injection

**Fig. 2:**
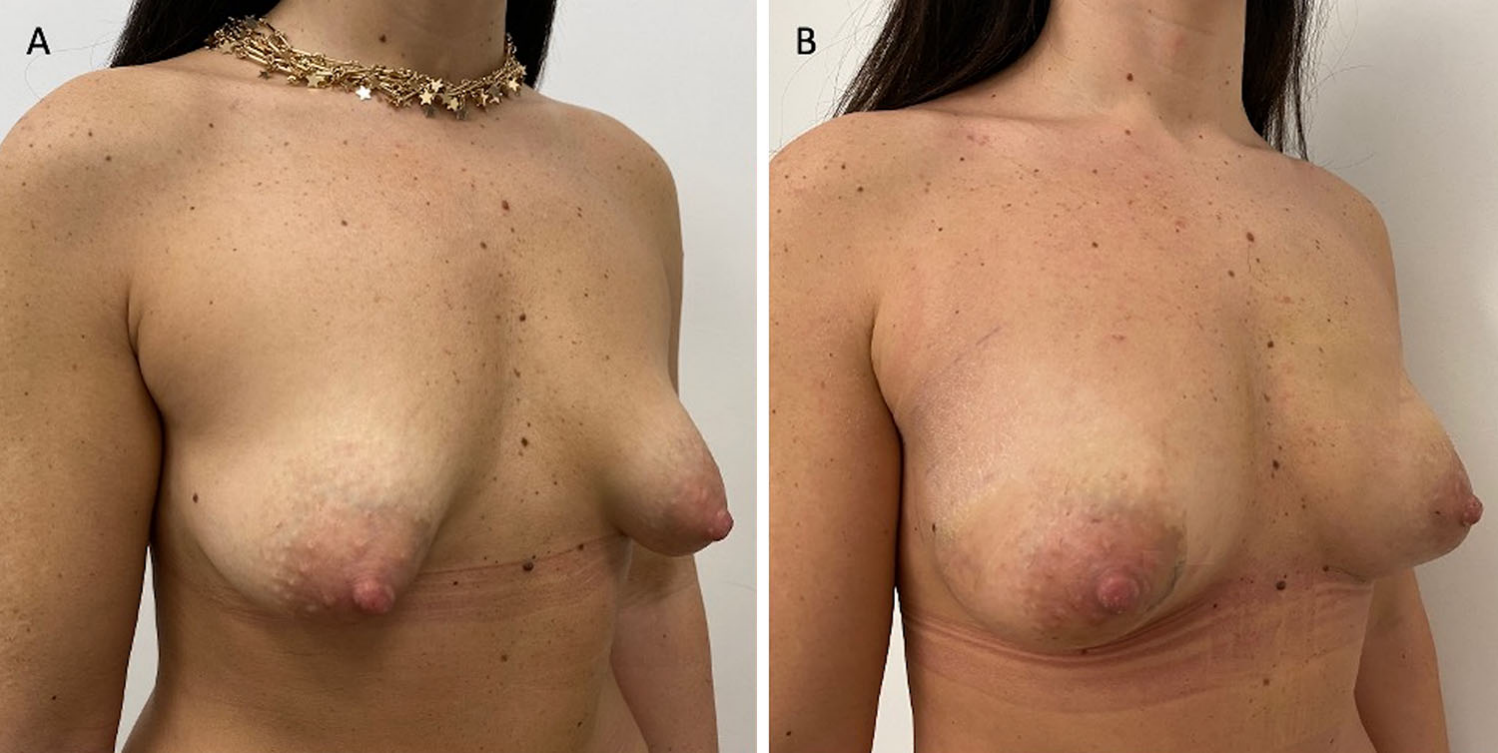
Same patient shown in Fig. 1. **A** ¾ right preoperative view showing hypoplasia of both LQs; **B** ¾ right postoperative view 12 months later only one fat grafting injection

**Fig. 3:**
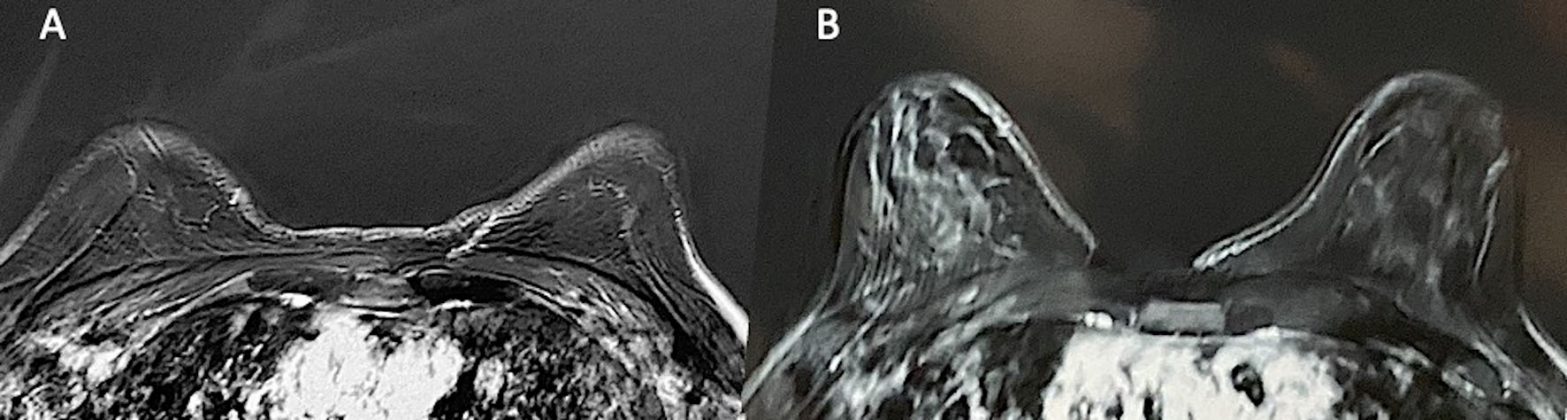
Bilateral Axial T2-weighted short tau inversion recovery MRI scans of the study group’s breasts. **A** Preoperative situation of the patient showed in Fig. 1. **B** Postoperative obtained 12 months later the only one fat graft session of patient-reported in Fig. 1, in which it is showed contrast uptake of the glandular tissue, and a great improvement in the bilateral breast volume

**Fig. 4:**
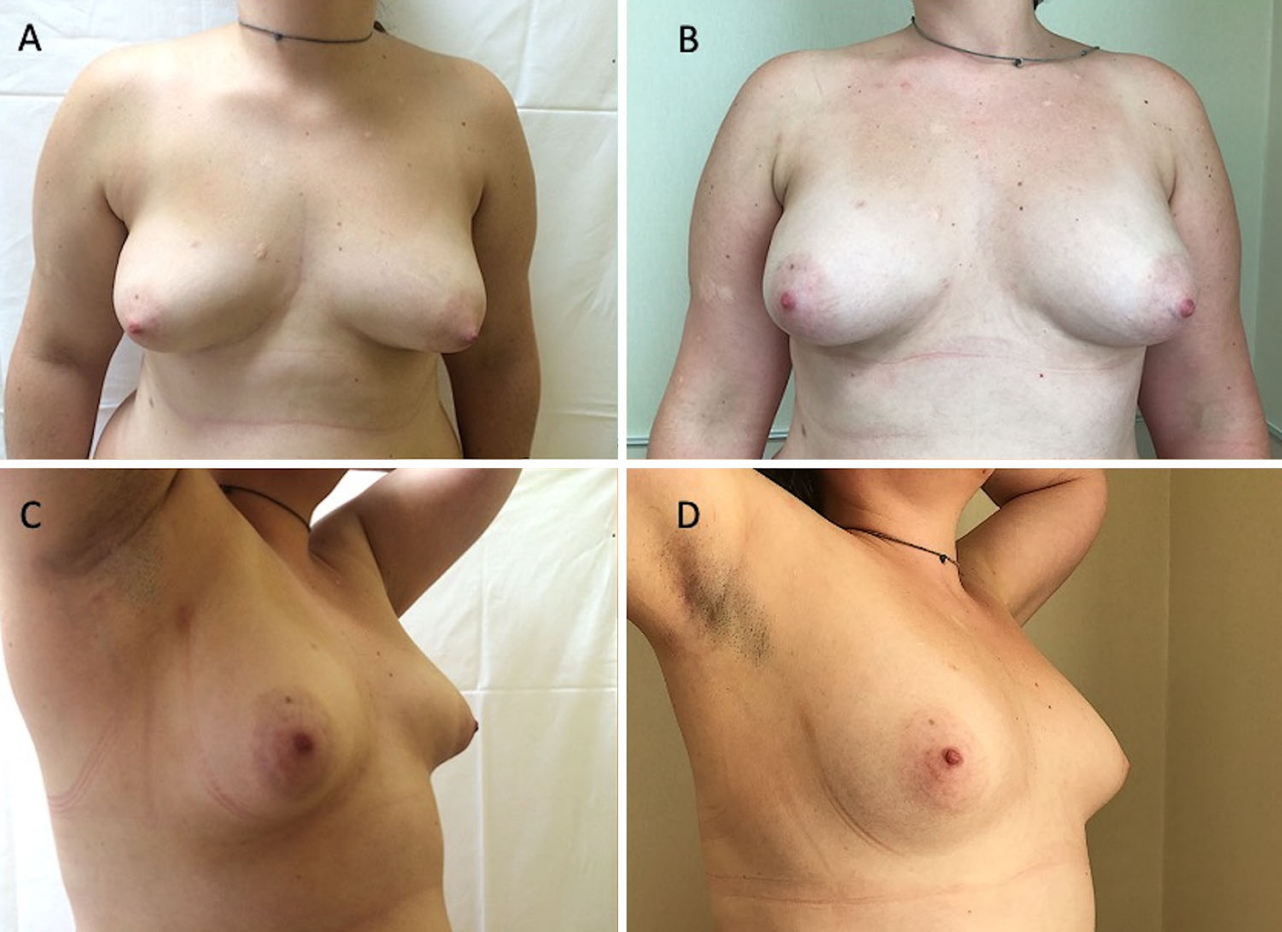
Analysis of study group’s patient, a 36-year-old female patient affected by a bilateral tuberous breast of type I and bilateral hypoplasia. **A** Frontal preoperative view showing hypoplasia of the medial lower quadrant; **B** Postoperative in frontal view 6 months later the second fat graft injection coinciding with T7. The second treatment was performed at T6. **C** ¾ right preoperative view; **D** ¾ right postoperative view 6 months later the second fat graft injection coinciding with T7

**Fig. 5:**
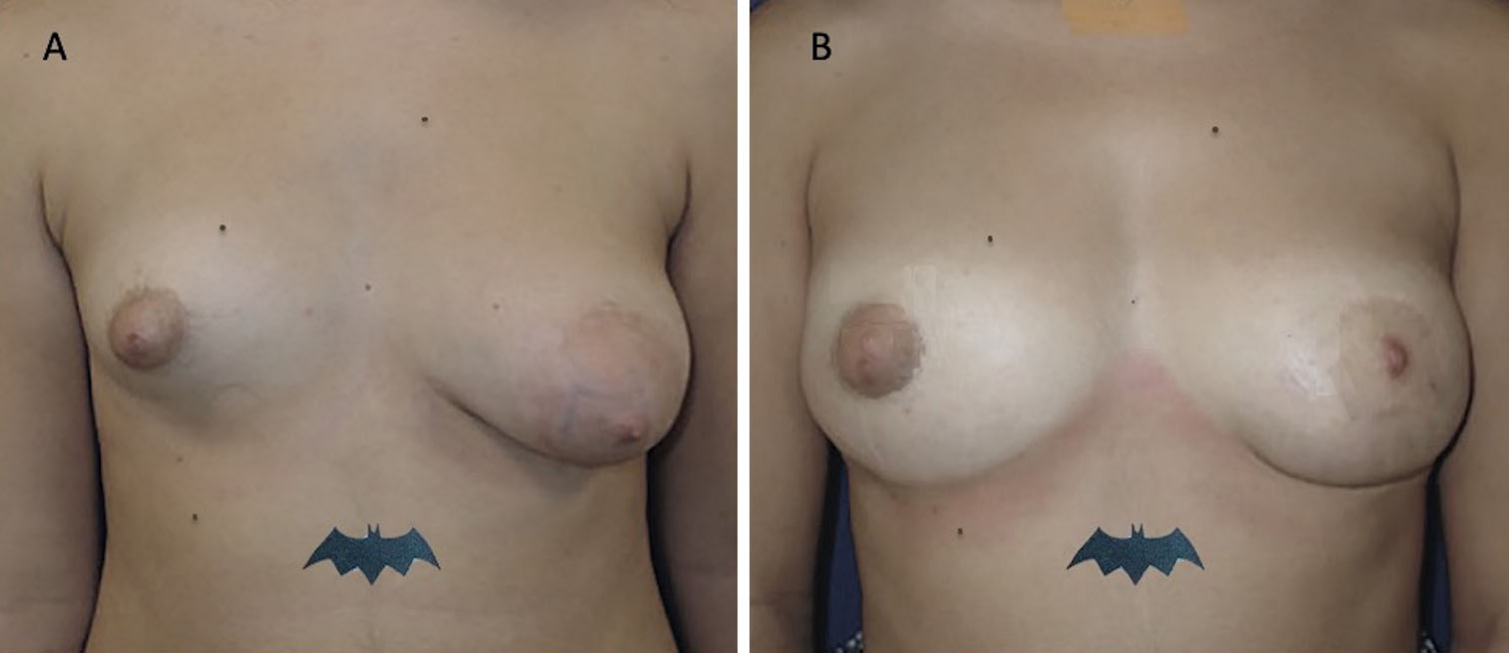
Analysis of the control group’s patient, a 29-year-old female patient affected by marked breast and nipple-areola complex asymmetries and unilateral tuberous breast. **A** Frontal preoperative view showing the right breast suffered from a high degree of hypoplasia while the left breast suffering from a unilateral tuberous breast of type II; **B** Postoperative in frontal view 12 months later peri-areolar mastopexy with implant (left) and definitive implant (right) with asymmetries and tuberosity correction

Thirty-five patients (34 females and 1 male) aged 18–60 years (average age 39) affected by breast deformity and/or tuberous breast with several degrees of breast hypoplasia and/or breast asymmetry (*n* = 10 with bilateral tuberous breast of type III and bilateral breast hypoplasia, *n* = 8 with a bilateral tuberous breast of type II and bilateral breast hypoplasia [Fig. 1A and Fig. 2A], *n* = 5 with unilateral tuberous breast of type II and unilateral breast hypoplasia with a high degree of breast asymmetry, *n* = 2 with breast deformity [included one male with Poland Syndrome] and a moderate degree of breast asymmetry (Fig. 3), and *n *= 10 suffering from the bilateral tuberous breast of type 1 with bilateral breast hypoplasia [Fig. 4A, C and Fig. 5A ])@ were treated with FG (study group - SG). Pre-menopausal patients were 26 (77%).

Thirty patients (all females) aged 18–63 years (average age 40.5), affected by breast deformity and/or tuberous breast with several degrees of breast hypoplasia and breast asymmetry (*n* = 7 with bilateral tuberous breast of type III and bilateral breast hypoplasia, n = 6 with a bilateral tuberous breast of type II and bilateral breast hypoplasia, *n* = 10 suffering from bilateral tuberous breast of type 1 with bilateral breast hypoplasia, *n* = 5 with unilateral tuberous breast of type II and unilateral breast hypoplasia with a high degree of breast asymmetry [Fig. 5A] and *n *= 2 with breast deformity and a moderate degree of breast asymmetry) were treated with M-SI procedure (control group - CG). Pre-menopausal patients were 21 (70%).

Areolar prolapse was noted on 10 tuberous breasts in the SG (29%) and on 8 tuberous breast in the CG (27%). Patients’ data and clinical assessment were detailed in Table 1.

**Table 1:** Patients’ data and clinical assessment

	FG Group (Study group)	M-SI Group (Control group)
Number of patients, no	35	30
Age at surgery, yr	39,0 (min 18, max 60)	40,5 (min 18, max 63)
BMI at surgery, kg/m2	26,5 (min 18, max 35)	26,5 (min 18, max 35)
Bilateral tuberous Breast with bilateral Hypoplasia Unilateral Tuberous Breast with unilateral breast hypoplasia Breast deformity Pre-menopausal Areolar prolapse Only one session Mean transfer volume for each breast after the first treatment Second session Mean transfer volume for each breast after the second treatment Total mean transfer volume for each breast Volume maintenance percentage after one treatment Volume maintenance percentage after a second treatment Cyst formation and Calcification Fat necrosis Double bubble Inadequate tuberous breast correction Capsular contracture Implant replaced for inadequate final volume	10 (type I), 8 (type II), 10 (type III) 5 (type II) + high degree of asymmetry 2 + moderate degree of asymmetry 26 (77%) 10 (29%) 8 (23%) 250mL (range 150–350mL) 27 (77%) at T6 Insufficient final volume re-treated with FG *n *= 27 180mL (range 100–260mL) 500 ml (range 250–750ml 68% (T6) 60% (T7) (All patients) 75% (T7) (All patients) 5 (14%) 0 0 0 0 0	10 (type I), 6 (type II), 7 (type III) 5 (type II) + high degree of asymmetry 2 + moderate degree of asymmetry 21 (70%) 8 (27%) 22 (73%) 285mL (range 150–420mL) 8 (27%) Double bubble *n *= 1; implant replaced *n *= 2; capsular contracture *n *= 2; re-treatment with FG injection *n *= 3 130 mL (range 80 ml180 ml) only in case of re-treatment with FG *n *= 3 285mL (range 150-420mL) 100% (All patients) 100% (All patients) 0 0 1 (3,5%) 2 (7%) 2 (7%) 2 (7%)

An accurate preoperative screening, based on a full clinical evaluation, a photographic and instrumental assessment performed by magnetic resonance imaging (MRI) (Fig. 3A), ultrasound, and mammography has been performed in all patients enrolled to estimate the type and entity of the deformity.

### Inclusion and Exclusion Criteria

Following inclusion criteria were considered: age 18-70 years old, history of tuberous breast with several degrees of breast hypoplasia, breast asymmetry, and breast deformity, Poland Syndrome, and a BMI between 18 and 35 kg/m^2^. Additional inclusion criteria in the M-SI group were (outcomes of previous breast surgeries) while in the FG group were sufficient fat in the sites of harvest. Following local and systemic exclusion criteria were considered: systemic (bone marrow aplasia, anti-aggregating therapy, sepsis, cancer, and uncompensated diabetes), local (reconstruction with expanders, reconstruction with the first stage FG followed by M-SI, cancer loss of substance, and uncontrolled comorbidities). Tobacco uses or genetic disorders were not considered exclusion criteria.

### Clinical Data Assessment and Quality Checks

Clinical data assessment and quality checks were based on the following criteria:

- Surveys related to the patient’s grade of satisfaction on resulting texture, softness, contours, and volume, availability to undergo the procedure again, to recommend the treatment to friends, and sufficient information about risks and complications (range 1–6: excellent [1]; very good [2]; good [3]; sufficient [4]; poor [5]; very poor [6]) (Appendix A);
- Clinical evaluation using the physician's overall assessment score (excellent, good, discreet, enough, poor, inadequate);
- Clinical evaluation using the patient’s overall assessment score (from excellent to inadequate);
- Visual Analog Scale (VAS) (range 1–10);
- Additional Factors/variables, such as breast asymmetry, breast deformity and chest deformities, NAC asymmetries, pseudoptosis, pectus excavatum and carinatum, low BMI, (presence: high, moderate, and low degree or absence);
- Adverse effects signaling (presence or absence).
- Patients were analyzed at 1, 2, 4, 8, 12, 24, 48, weeks, and then annually for 2 years by clinical examination while at 1 month (*T*3), 6 months (*T*6), 12 months (*T*7), and then annually until the second year later the last procedure by MRI and ultrasound. Abnormal clinical findings were further investigated.

### Breast Reconstruction with Mastopexy and/or definitive implants (M-SI): Surgical procedures

In the M-SI group, the SI has been positioned in 70% sub-glandular (21 patients) and 30% sub-muscular (9 patients). When simple augmentation was not sufficient to re-establish symmetry (e.g., tuberous breast and deficit of the lower pole) the Ribeiro [16] technique of glandular reshape was used. The surgical incisions adopted in the M-SI group were inframammary (40%–12 patients) and peri-areolar (60%–18 patients). All M-SI group patients received round SI. The size (between 150 and 420 ml with a mean of 285 ml), profile (moderate or high), and surface (smooth or textured) of the prosthesis have been decided according to preoperative tuberous breast type (I, II, III), breast asymmetry’ degree (high, moderate, or low), presence of unilateral or bilateral tuberous breast, breast' tissue thickness, and patient expectations. If the patient had nipple asymmetry (in the case of vertical or horizontal NAC malposition) or had tuberous breasts with hypoplasia, a peri-areolar incision was used (6 bilateral tuberous breast type II, 7 bilateral tuberous breast type III, 5 unilateral tuberous breast type II). In patients where only SI positioning was sufficient (without mastopexy), an inframammary incision was used (10 bilateral tuberous breast type I, 2 breast deformity). Initially, the surgeon’s preference was the smooth prosthesis for dual-plane pockets and textured implants for sub-glandular pockets. Currently, smooth implants for both are used. The choice of the SI and the related positioning plane was performed based on the tissues available, with a “tailor-made” approach. In all cases, drains were applied. Vertical and radial cutting scoring of the inferior gland was performed if needed to expand the breast inferior segment or in all cases of tuberous breast. Any evident abnormality in breast volume (high grade of breast hypoplasia or unilateral breast hypoplasia), NAC position (vertical asymmetry), breast shape (tuberosity type III or unilateral tuberous breast with a high degree of breast asymmetry), or the presence of pseudoptosis, very thin patient, pectus excavatum and carinatum were defined as a non-optimal result, here identified, and called “suboptimal result”. Deformities correlation coefficient (DCC) was also calculated to their result influence. The patient’s clinical features and deformities have been deeply analyzed aiming to identify a list of factors that could have downgraded the outcome of the procedure and identify the incidence of each of these deformities. The number of preoperative deformities for each patient was determined, and the relationships between the suboptimal results and these deformities were evaluated. Preoperative deformities were considered complicating factors and were then classified into major or minor factors. Major complicating factors (M-CFs) were those that singularly led to a suboptimal outcome, and minor factors (m-CFs) were those that led to a suboptimal outcome if they were present in combination (Table 2).

**Table 2:** Complicating factors classification

Deformity	FG group	M-SI group
Major complicating factors (M-CFs)	Vertical NAC asymmetry *n* = 3	Vertical NAC asymmetry *n *= 2
	Pectus excavatum *n *= 4 Pectus carinatum *n *= 1 – Breast deformity *n *= 2	Pectus excavatum *n *= 3 Pectus carinatum *n *= 1 Low BMI *n *= 1 Breast deformity *n *= 2
Minor complicating factors (m-CFs) (combing of two)	High degree of asymmetry + unilateral tuberous breast + pseudoptosis *n *= 7	High degree of asymmetry + unilateral tuberous breast + pseudoptosis *n *= 5
Minor complicating factors (m-CFs) (only one)	Scoliosis + Horizontal NAC asymmetry *n* = 2 Scoliosis *n *= 2 thin skin *n *= 2 Horizontal NAC asymmetry *n *= 2	Scoliosis + Horizontal NAC asymmetry *n *= 1 Scoliosis *n *= 2 thin skin *n *= 1 Horizontal NAC asymmetry *n *= 2

### Breast Reconstruction with Fat Grafting (FG): Surgical Infiltration

Based on the acquired MRI scans, volumetric fat site assessments into the breasts were calculated, utilizing as edges the anterior axillary line, anterior margin of the pectoral muscle, medio-sternal line, skin, and nipple employing a 3D reconstruction.

Every breast was considered like a geometric “cone” and for this reason, the formula Volume = π × r^2^ × h / 3 (base area x-height, divided 3) to evaluate the breast’s initial volume and the optimal volume of fat to inject it has been applied [9], so to permit the injection of the same amount of fat in milliliters corresponding to the initial volume expressed in cm^3^ [9].

The FG infiltration was performed using the "Gentle-technique” [17] based on slow and controlled movements, implanting linear deposits of FG in subcutaneous tissue–supra-fascial, retro-glandular, and intra-glandular–(not into pectoralis major muscle), through multiple tunnels and seven different entrances (inframammary fold [located at 130°,180°,220°], higher external quadrant [290°], lower-external quadrant [240°], higher internal quadrant [65°] and lower-internal quadrant [110°]) using 1.5mm cannulas [17, 18].

### Minimal Manipulation of Fat Grafting (FG)

The FG was prepared according to minimal manipulations rules prevalently based on centrifugation and filtration, commonly considered mechanical digestion. Also, enzymatic digestion of fat tissue may be considered a minimal manipulation in agreement with the reflection paper EMA/CAT/600280/2010 Rev 1, 20 June 2014, by the CAT, only when “leads to isolation of functionally intact tissue or cells maintaining the original function when they are used in a similar anatomical site or histological condition” and when the tissue/cells obtained“ are used with the same function for an in donor & recipients without any changing of their biological characteristics”.

**Fig. 6:**
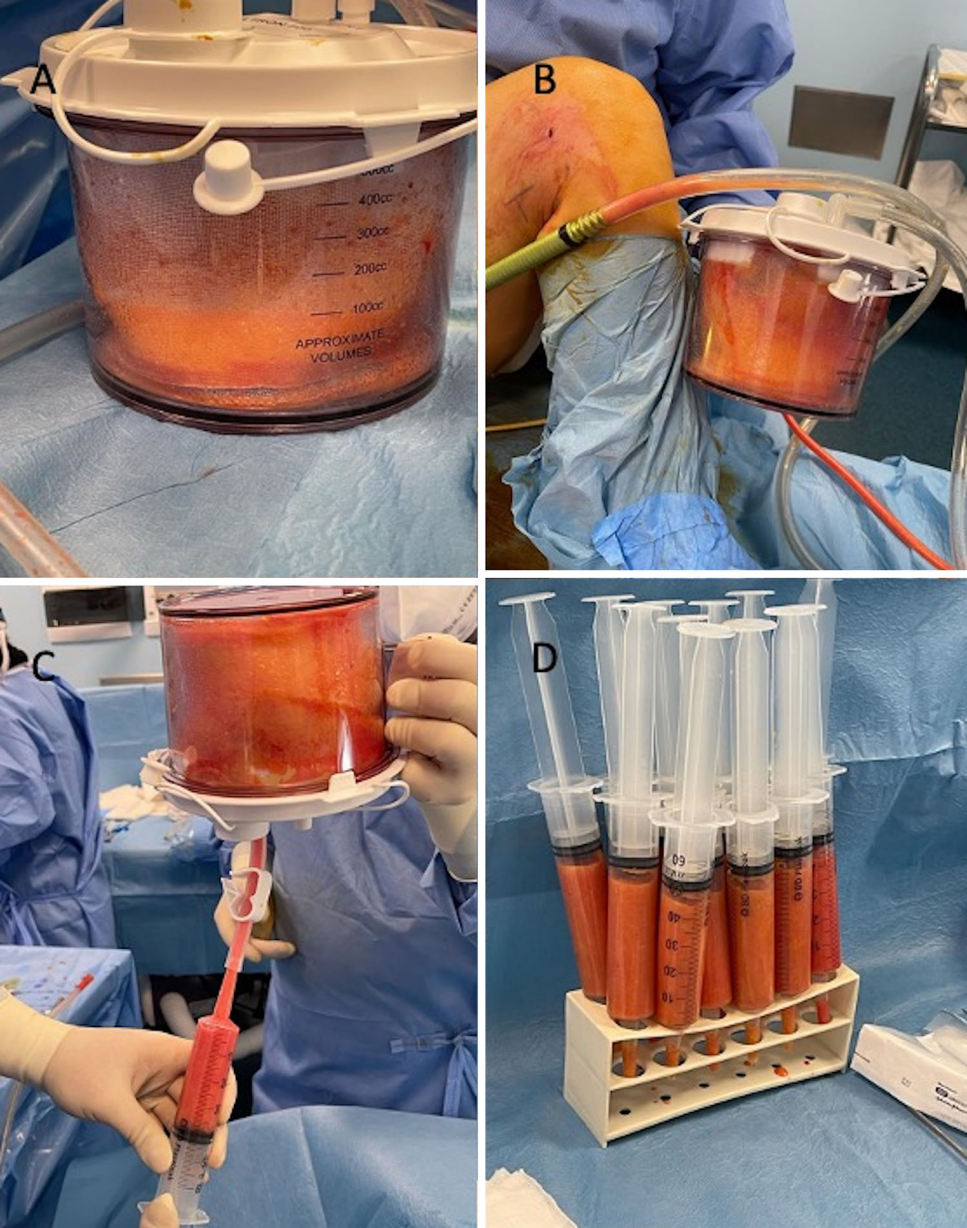
The mechanical digestion of fat tissue, based on filtration procedures, using Tissu-Trans Filtron^®^ - Class IIa product- (Summit Medical, LLC 815 Vikings Parkway Suite 100 Saint Paul, MN 55121 USA, https://shippertmedical.com/products/tissu-trans-filtron-250). **A** The Tissu-Trans Filtron 500mL closed inline filtration system during the harvesting; **B** Fat (800 mL average in all patients—range 400mL/1200 mL) was collected and purified directly without any procedure of centrifugation, using a standard suction connected directly to the canister permitting to filtering non-viable cells during harvest, saving valuable surgical time; **C** Purified fat harvesting, using 60mL syringes; **D** Ten 60mL syringes charged to 50mL (for a total of 500mL of purified fat grafting are ready to be used

- The enzymatic digestion was performed on 13 patients (37%) using Celution™ 800/CRS System (Cytori Therapeutics Inc., San Diego, CA, USA, http://www.cytoritx.com) obtaining within 160 min, a 365 mL average of ASCs-enhanced fat tissue, ready for transplantation, as previously reported [8–10, 12].
- The mechanical digestion was performed on 22 patients (63%) using Tissu-Trans Filtron^®^ - Class IIa product- (Summit Medical, LLC 815 Vikings Parkway Suite 100 Saint Paul, MN 55121 USA, https://shippertmedical.com/products/tissu-trans-filtron-250) and Fatstkit (CORIOS Soc. Coop, San Giuliano Milanese, Italy, https://www.cylex-italia.it/san-giuliano-milanese/corios--soc-coop-r-l---commercio-presidi-chirurgici-13161214.html). In the first case, using the Tissu-Trans Filtron^®^ 500mL closed inline filtration system (Fig. 6A), fat (800 mL average in all patients— range 400mL/1200 mL) was collected and purified directly without any procedure of centrifugation, using a standard suction connected directly to the canister (Fig. 6B), permitting to filtering non-viable cells during harvest, saving valuable surgical time. At the end of the harvesting, an average of 400 mL of purified fat (range 200mL/600mL) ready to be injected was obtained (Fig. 6C, D). The fat harvesting and purification process—performed at the same time—were completed within 90 min. In the second case, using Fatstkit, an amount of fat (480 mL average) was purified and enriched with ASCs within 120 min, after centrifugation and filtration procedures [12].

### Statistical Analysis

A comparison between M-SI and FG groups was done with the Student’s *t* test or Mann–Whitney for the question of the self-assessment questionnaire. The data are expressed by mean (range), median (range), and percentages. A two-tailed *p*-value less than 0.05 has been identified as significant.

## Results

### Clinical Assessment

**Fig. 7:**
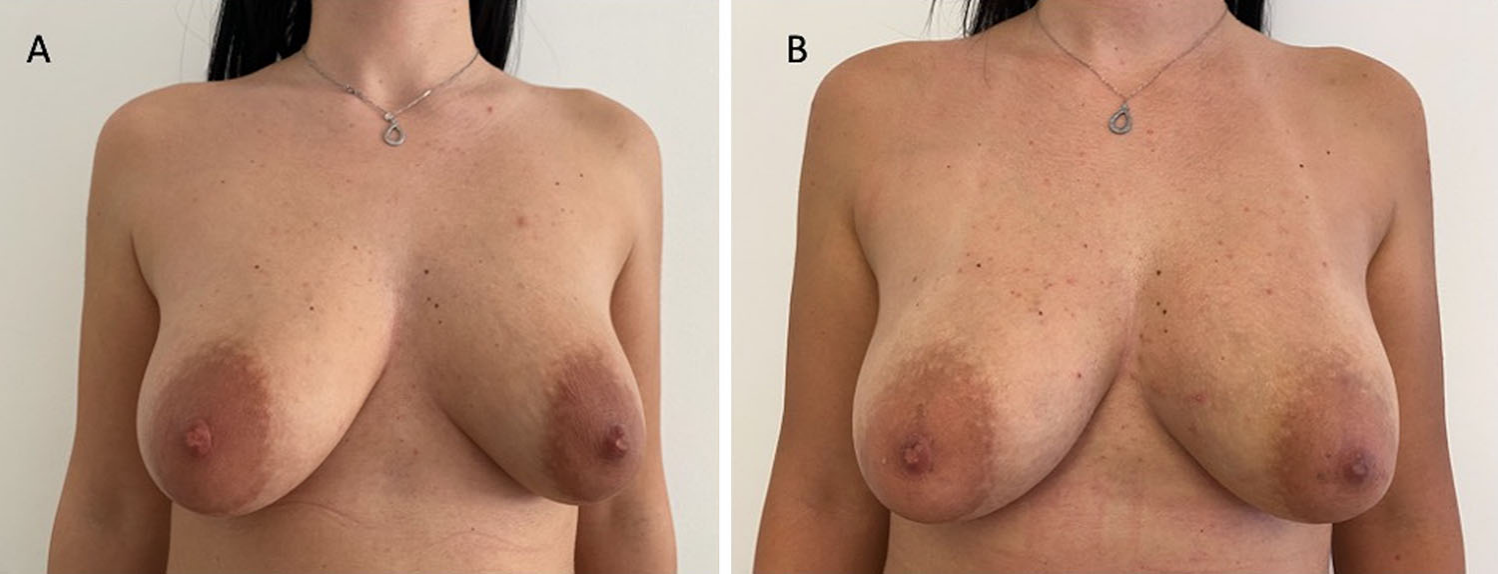
Analysis of the study group’s patient, a 27-year-old female patient affected by a bilateral tuberous breast of type I. **A** Frontal preoperative view showing only hypoplasia of the medial lower quadrant; **B** Postoperative in frontal view 12 months later only one fat grafting injection

Excellent results characterized both by an improvement in the breast shape (in the lower pole and all anatomic areas affected by tuberosity) and by volume increase have been observed in 77% (*n *= 27) of the patients with tuberous breasts treated “two times” with FG (*p *= 0.475 vs. M-SI group). In detail, an increase of 38.5mm in the three-dimensional (3D) breast volume after 1 month (T3), 27.1mm after 6 months (T6), and 21.7mm after 12 months (T7) (Fig. 1B, Fig. 2B, Fig. 7B), analyzed by MRI and by clinical comparison between pre-and post-op were observed after only one FG infiltration (Fig. 3A, B). The second FG injection was performed at T6 in 27 patients (77%) and an additional increase of 32.5mm in the 3D volume was observed at 1 year (T7) (Fig. 4B, D), with a total volume increase of 71.0mm analyzed by MRI with 3-mm-thick slices, evidencing a result comparable with that obtained by the SI.

Patients treated with only one FG showed 68 and 60% volume maintenance, respectively, at T6 (6 months later) and T7 (after 1 year and without the second FG). Patients treated with a second FG at T6 showed 75% volume maintenance after 6 months coinciding with T7 (Fig. 4B, D). FG resorption was analyzed with instrumental MRI and ultrasound.

Excellent results with the above-mentioned characteristics were observed in 73% (*n* = 22) of patients with tuberous breasts treated “one time” with M-SI at T7 (after 12 months) (Fig. 5B). The breast contour restoring maintenance in the M-SI group was higher than that in the FG group (*p *< .0001 *vs.* FG group) thanks to the use of SI. More natural results in the FG group were higher than that in the M-SI group (*p* < .0001 *vs.* M-SI group). The patient’s satisfaction in the FG group was higher than that in the M-SI group (*p *< .0001 *vs.* M-SI group) also thanks to the absence of scars.

A comparable result to that of M-SI at 1 year (T7) has been obtained in the FG patients treated with “only one” FG infiltration in 23% (*n* = 8) of cases (Fig. 1B, Fig. 2B).

Both FG and M-SI patients referred to satisfaction with the resulting texture, softness, and volume contours, additionally a major part of people was satisfied with the outcomes obtained (*p* = 0.425) in terms of tuberous breast, breast asymmetry, and breast hypoplasia correction, making themselves available to undergo the procedure again (*p* > 0.753), and recommending the treatment to friends (*p* = 0.326) (Table 3). Regarding the self-evaluation of tuberosity’ correction after 1 year and one treatment, scores ranged from 3 to 6 in the FG group and from 1 to 4 in the M-SI group (*p* = 0.033) displaying more satisfaction in the M-SI group than FG group (Table 3). The trend appeared to overlap when the self-evaluation was performed at T7 in FG patients treated two times, showing a score ranging from 1 to 4. Satisfaction grade assessment questionnaire analysis displayed that all patients in both groups (FG and M-SI) would choose to undergo FG or M-SI, and they were fully informed about the risks and complications of these treatments (including the risk of FG resorption, the high possibility to repeat the treatment more times in FG group and the risk of visible scars and prosthesis displacement or reject in M-SI group).

**Table 3:** Patient satisfaction data

	FG Group	M-SI Group
Patients no	35	30
Self-evaluation of reconstructive results after “one treatment” (score range 1-6 / excellent-very poor) Self-evaluation of reconstructive results after “two treatments” (score range 1-6 / excellent-very poor)	15 (Fully satisfied): 8 (Excellent/1) 4 (Very good/2) 3 (Good/3) 6 (Not/Fully/satisfied): 6 (Sufficient/4) 9 (Not/satisfied): 6 (Poor/5) 3 (Very poor/6) 33 (Fully satisfied): 27 (Excellent/1) 4 (Very good/2) 2 (Good/3) 1 (Not/Fully/satisfied): 1 (Sufficient/4) 1 (Not/satisfied): 1 (Poor/5) 0 (Very poor/6)	22 (Fully satisfied): 22 (Excellent/1) 0 (Very good/2) 0 (Good/3) 3 (Not/Fully/satisfied): 3 (Sufficient/4) 5 (Not/satisfied): 4 (Poor/5) 1 (Very poor/6) 29 (Fully satisfied): 23 (Excellent/1) 6 (Very good/2) 0 (Good/3) 1 (Not/Fully/satisfied): 1 (Sufficient/4) 0 (Not/satisfied): 0 (Poor/5) 0 (Very poor/6)
Satisfaction of final result Recommend the treatment to a friend Available to breast reconstruction Sufficiently informed on risks and complications	34 34 35 35	29 29 30 30

When satisfaction grade was evaluated via VAS, people of FG and M-SI groups were similarly satisfied (*p* = 0.23). Figures. 1, 2, and 7 show patients that were categorized as showing “improvement” by all peers. When computing the new scores, patients in the M-SI group and the FG group received the respective scores (average) of 4.8 and 2.9 (*p *= 0.32) and, therefore, were regarded as presenting better improvement in the M-SI group. Additionally, the absence of a peri-areolar scar and/or inframammary fold scar (5 cm average) in the FG group appeared fundamental in the outcomes. As expected, patient satisfaction with the appearance of the scar was higher in the FG group, in which the scars were 2 mm in maximum diameter.

### Breast and Chest Deformity Influence Reconstructive Outcomes

Breast deformity and/or chest deformity were detected in 25 FG patients (72%) (10 patients with M-CFs, 9 with a combination of two or more m-CFs, and 6 with only one m-CF) and 20 M-SI patients (67%) (9 patients with M-CFs, 6 with a combining of two or more m-CFs and 5 with only one m-CF).

Eight patients of the M-SI group had suboptimal results compared with only 4 FG patients. The deformities influenced more negatively the M-SI group outcomes than the FG group (DCC = 0.083, *p *< .001).

### Study Limitations

Performing a deep analysis of the results obtained during this investigation, several limitations led to the presence of bias in the present work. Firstly, regarding the FG group, a lack of a single standardized protocol for the isolation methods of ASCs has been highlighted, as well as standardized enrichment or purification procedures. A total of three procedures, two based on enrichment of FG performed by mechanical ASCs isolation (centrifugation with filtration), and the digestion method (enzymatic), while one based on purification (without enrichment or centrifugation) have been analyzed. This difference may influence the fat maintenance percentage. Secondly, regarding the M-SI group, the differences in terms of implant positioning (sub-muscular or sub-glandular), and the incision (peri-areolar or peri-areolar and vertical, or inframammary) makes the group not homogeneous.

In each case, the bias has been limited by the "tailor-made approach" for every patient, in which the choice of the incision during mastopexy, definitive prosthesis, and the related positioning plane was performed based on the degree of the TB (I -no mastopexy only inframammary fold incision for breast implanting; II – periareolar incision + SI; III – periareolar and vertical incision + SI), tissues available (sufficient gland=sub glandular plane / very thin thickness grade of the gland and related tissues=sub-muscular plane / low thickness grade of glands and soft tissues=dual plane).

The present investigation appears as the first retrospective case-control study in this field, and for this reason, additional perspectives study and/or controlled trials will be necessary.

## Discussion

Tuberous breast deformity represents a challenge for the plastic surgeons starting by its classification proposed by several authors. Diversely by the Grolleau et al. [1] that classified the tuberous breast in three different types (I-III) as already reported, Mandrekas and Zambacos [29] affirmed there are only two constricted areas at the tuberous breast deformity affecting the severity, one is at the base of the breast and the other is behind the NAC. On this way, several techniques have been proposed to correct this deformity, also depending by its severity, and several papers in the literature discussed the use of prosthetic and autologous treatments for breast asymmetry [19]. Some studies favor SI over FG, while others underline the advantages of FG in terms of esthetic result, natural appearance, and stability of result over time [10]. The FG injection may also not solve the tuberous deformity since part of the issue includes minimizing the herniation of the breast gland. This study contributes to the analysis of outcomes for a large patient cohort (65 patients) with similar asymmetries and deformities features. The data here reported showed a similar outcome after "two fat injections" *vs* "one implant surgery" (77% vs. 73% of excellent results) (*p *= 0.475 vs. M-SI group). M-SI group showed has better satisfaction with only one surgery, while in the case of the FG group, the patient achieved the same satisfaction without the use of implants after two surgeries. These data suggest that FG could be considered the mainstay procedure in treating complex breast asymmetry cases, unilateral tuberous breast, and/or cases of chest deformity associated. Breast asymmetry appears as the most common issue found. Its incidence has been reported to be 88% by Rohrich et al [20] (*n *= 100) and 81.1% by DeLuca-Pytell et al [21] (*n* = 375). The analysis of M-CFs and m-CFs has been done according to the implication degree and related effect produced by a specific deformity on the outcomes. In the present study, M-CFs have been defined as deformities that could, if presented alone, lead to a suboptimal result, while the m-CFs were those that led to a suboptimal outcome only if they were combined. A single m-CF has less of an effect on the outcome and might even pass unnoticed if not corrected. It is necessary to highlight suboptimal results, in the present study, were not defined based on patient satisfaction because there were satisfied patients with suboptimal results represented by 15 SI patients and 19 FG patients that, respectively, presented one of the M-CF or a combination of two m-CFs. Some papers show that patients with high or low BMI have more postoperative side effects [22, 23]. In the current study, patients with low BMI presented diminished skin envelope, making the treatment challenging both for FG and M-SI. Pectus excavatum is another deformity that can get worse outcomes especially when M-SI was used. The right option for camouflaging this deformity is the FG. Scoliosis deformity, even if subtle, may present as breast asymmetry [24]. There is a correlation between the severity of scoliosis and the difference in breast volumes [25]. The NAC may be asymmetric on both the vertical and horizontal axis. A NAC asymmetrically placed on the horizontal axis was firstly reported by Khan [26], describing a related incidence of a 12% rate.

When satisfaction grade was evaluated through the VAS, people of M-SI and FG groups were similarly satisfied (*p* = 0.23), showing results in-line with those reported by Brault et al [19], albeit with some difference. Brault et al [19] compared complications and satisfaction in 37 patients (analyzed by Breast-Q scale) suffering from tuberous breast deformity and treated either with FG (27 breasts) or SI (36 breasts). Here the results have been obtained in a cohort of 65 patients, and diversely by Brault et al. [19], the patients’ satisfaction grades were better in the FG group (after two procedures) also thanks to the scars’ absence and more natural results versus the M-SI group.

Several studies agree that FG is becoming the routine treatment for this kind of deformity as it avoids the complications related to the placement of the implant and responds physiologically to the breast changes over time [4, 27].

FG performed to improve breast asymmetry appears advantageous in several ways: (I) the purely autologous nature of the operation, (II) the reproducibility, (III) the natural appearance and consistency of the breast postoperatively, (IV) the postoperative symmetry with the contralateral breast, and finally (V) the secondary benefits of the liposuction involved in the procedure [5, 28].

## Conclusions

Complicating factors in tuberous breast treatment must be identified during the preoperative evaluation aiming to recognize challenging cases and plan a more adequate surgical procedure. Breast deformities (asymmetry and tuberous breast with high grade of hypoplasia, unilateral breast hypoplasia, NAC asymmetry), chest deformities (pectus excavatum and carinatum), and low BMI may compromise the outcome, being M-CFs. FG treatment determined more natural results, allowing also to treat with better results compared with M-SI, patients with pectus excavatum and/or carinatum, volume asymmetry, and unilateral breast hypoplasia. M-SI treatment determined a larger breast volume and excellent results after a single procedure (compared to the two necessaries for FG). In order to more accurately assess and comprehend the postoperative findings linked to patient satisfaction, a prospective study seems to be required in the near future.

## Appendix 1. Patient’s satisfaction questionnaire

Degree of Satisfaction:

1. 1 - Excellent;
2. - Very good;
3. – Good;
4. – Sufficient;
5. – Poor;
6. – Very poor.

In the preoperative period

According to the scale above, grade questions 1 through 6 according to your degree of satisfaction:

1. How do you feel about your breast shape?
2. How do you feel about your breast volume?
3. How do you feel about your breast symmetry?
4. How do you feel about your breast softness?
5. How do you feel about your breast texture?
6. Did you have any pieces of information on risk and complications (including the risk of reabsorption of fat graft and the high possibility to repeat the treatment more times)?

In the postoperative period at 1 week (T1), 2 weeks (T2), 4 weeks (T3), 8 weeks (T4), 3 months (T5), 6 months (T6), 12months (T7), and then annually.

According to the scale above, grade questions 1 through 6 according to your degree of satisfaction:

1. How do you feel about your breast shape?
2. How do you feel about your breast volume?
3. How do you feel about your breast symmetry?
4. How do you feel about your breast softness?
5. How do you feel about your breast texture?
6. Did you have any pieces of information on risk and complication (including the risk of reabsorption of fat graft and the high possibility to repeat the treatment more times)?

## References

[1] Grolleau JL, Lanfrey E, Lavigne B (1999). Breast base anomalies: treatment strategy for tuberous breasts, minor deformities, and asymmetry. Plast Reconstr Surg.

[2] Brown MH, Somogyi RB (2015). Surgical strategies in the correction of the tuberous breast. Clin Plast Surg.

[3] Pacifico MD, Kang NV (2007). The tuberous breast revisited. J Plast Reconstr Aesthet Surg.

[4] Claudio Silva-Vergara C, Fontdevila J, Weshahy O (2018). Fat grafting technique, a paradigm shift in the treatment of tuberous breast. World J Plast Surg.

[5] Delay E, Guerid S (2015). The role of fat grafting in breast reconstruction. Clin Plast Surg.

[6] Nahabedian MY (2011). Breast deformities and mastopexy. Plast Reconstr Surg.

[7] Rees TD, Aston SJ (1976). The tuberous breast. Clin Plast Surg.

[8] Gentile P, Casella D, Palma E (2019). Engineered fat graft enhanced with adipose-derived stromal vascular fraction cells for regenerative medicine: clinical, histological and instrumental evaluation in breast reconstruction. J Clin Med.

[9] Gentile P, Kothari A, Casella D (2020). Fat graft enhanced with adipose-derived stem cells in aesthetic breast augmentation: clinical, histological, and instrumental evaluation. Aesthet Surg J.

[10] Gentile P (2021). Breast silicone gel implants versus autologous fat grafting: biomaterials and bioactive materials in comparison. J Clin Med.

[11] Bayram Y, Zor F, Karagoz H (2016). Challenging breast augmentations: the influence of preoperative anatomical features on the final result. Aesthet Surg J.

[12] Gentile P, Scioli MG, Orlandi A (2015). Breast reconstruction with enhanced stromal vascular fraction fat grafting: what is the best method?. Plast Reconstr Surg Glob Open.

[13] Araco A, Gravante G, Araco F (2006). Breast asymmetries: a brief review and our experience. Aesthetic Plast Surg.

[14] Schuklenk U, Ashcroft R (2000). International research ethics. Bioethics.

[15] Von Elm E, Altman DG, Egger M (2008). The strengthening the reporting of observational studies in epidemiology (STROBE) statement: guidelines for reporting observational studies. J Clin Epidemiol.

[16] Ribeiro L, Canzi W, Buss A (1998). Tuberous breast: a new approach. Plast Reconstr Surg.

[17] Gentile P, De Angelis B, Di Pietro V (2018). Gentle is better: the original gentle technique for fat placement in breast lipofilling. J Cutan Aesthet Surg.

[18] Petit JY, De Lorenzi F, Rietjens M (2007). Technical tricks to improve the cosmetic results of breast-conserving treatment. Breast.

[19] Brault N, Stivala A, Guillier D (2017). Correction of tuberous breast deformity: a retrospective study comparing lipofilling versus breast implant augmentation. J Plast Reconstr Aesthet Surg.

[20] Rohrich RJ, Hartley W, Brown S (2003). Incidence of breast and chest wall asymmetry in breast augmentation: a retrospective analysis of 100 patients. Plast Reconstr Surg..

[21] DeLuca-Pytell DM, Piazza RC, Holding JC (2005). The incidence of tuberous breast deformity in asymmetric and sym-metric mammaplasty patients. Plast Reconstr Surg.

[22] Chen CL, Shore AD, Johns R (2011). The impact of obesity on breast surgery complications. Plast Reconstr Surg.

[23] Valente DS, Zanella RK, Doncatto LF (2014). Incidence and risk factors of striae distensae following breast augmentation surgery: a cohort study. PLoS ONE.

[24] Fredricks S (1978). Skeletal and postural relations in augmentation mammaplasty. Ann Plast Surg.

[25] Tsai FC, Hsieh MS, Liao CK (2010). Correlation between scoliosis and breast asymmetries in women undergoing augmentation mammaplasty. Aesthetic Plast Surg.

[26] Khan UD (2009). Breast augmentation in asymmetrically placed nipple-areola complex in the horizontal axis: lateralization of implant pocket to offset lateralised nipples. Aesthetic Plast Surg.

[27] Ho Quoc C, Piat JM, Michel G (2015). Fat grafting to improve severe tuberous breast. J Gynecol Obstet Biol Reprod.

[28] Largo RD, Tchang LA, Mele V (2014). Efficacy, safety and complications of autologous fat grafting to healthy breast tissue: a systematic review. J Plast Reconstr Aesthet Surg.

[29] Mandrekas AD, Zambacos GJ (2010). Aesthetic reconstruction of the tuberous breast deformity: a 10-year experience. Aesthet Surg J.

